# Semantics-Assisted Training Graph Convolution Network for Skeleton-Based Action Recognition

**DOI:** 10.3390/s25061841

**Published:** 2025-03-15

**Authors:** Huangshui Hu, Yu Cao, Yue Fang, Zhiqiang Meng

**Affiliations:** College of Computer Science and Engineering, Changchun University of Technology, Changchun 130012, China; huhuangshui@ccut.edu.cn (H.H.); 2202103047@stu.ccut.edu.cn (Y.F.); 2202203011@stu.ccut.edu.cn (Z.M.)

**Keywords:** action recognition, semantic relationships, semantics-assisted training, feature fusion

## Abstract

The skeleton-based action recognition networks often focus on extracting features such as joints from samples, while neglecting the semantic relationships inherent in actions, which also contain valuable information. To address the lack of utilization of semantic information, this paper proposes a semantics-assisted training graph convolution network (SAT-GCN). By dividing the features outputted by the skeleton encoder into four parts and contrasting them with the text features generated by the text encoder, the obtained contrastive loss is used to guide the overall network training. This approach effectively improves recognition accuracy while reducing the number of model parameters. In addition, angle features are incorporated into the skeleton model input to aid in classifying similar actions. Finally, a multi-feature skeleton encoder is designed to separately extract features such as joints, bones, and angles. These extracted features are then integrated through feature fusion. The fused features are then passed through three graph convolution blocks before being fed into fully connected layers for classification. Extensive experiments were conducted on three large-scale datasets, NTU RGB + D 60, NTU RGB + D 120, and NW-UCLA to validate the performance of the proposed model. The results show that the SAT-GCN outperforms others in terms of both accuracy and number of parameters.

## 1. Introduction

In today’s digital era, there is a growing demand for human behavior recognition across diverse domains including security [1], healthcare [2], virtual reality [3], transportation [4], and beyond. Human skeleton behavior recognition, a vital area within computer vision and pattern recognition, seeks to identify various actions or behaviors performed by individuals by analyzing the motion patterns of key points on the human body. However, achieving precise human skeleton behavior recognition remains challenging due to the intricate and varied nature of human skeleton data, as well as the complexities involved in understanding action semantics.

Traditional methods for human skeleton behavior recognition typically rely on handcrafted feature extractors and classifiers, which are limited by the expressiveness and generalization ability of the features. In recent years, with advancements in deep learning techniques, skeleton-based approaches [5,6,7] have emerged, leveraging graph convolutional models to represent the spatiotemporal features of skeleton joints and bones. Graph convolutional networks (GCNs) effectively exploit spatial and temporal relationships among human joints, thereby enhancing the feature representation capability of skeleton data. However, skeleton-based methods face constraints due to the absence of texture and appearance features. In datasets like NTU RGB + D 60 [8], where there are pairs of actions with similar skeletal motions such as “writing” and “typing”, “reading” and “writing”, or “putting on shoes” and “taking off shoes”, distinguishing between them using skeleton-based methods becomes challenging.

Most skeleton-based action recognition networks typically focus on extracting features such as joints, bones, and velocities from samples, because these features are crucial for capturing the fundamental motion patterns and dynamic characteristics of actions. However, these networks often overlook the semantic relationships of actions, which also contain rich information. For example, when identifying actions like “drinking water” and “eating”, although they may exhibit similarities in joint and skeletal movements, their primary differences may lie in hand gestures, such as picking up a cup or using utensils. This semantic information can better reveal the differences between them, thus helping the model to more accurately discriminate between actions. Therefore, effectively leveraging semantic information has the potential to significantly improve the recognition performance of the model.

### Contributions

This paper introduces a semantics-assisted training graph convolution network (SAT-GCN), which utilizes a text encoder to extract textual descriptions from action categories for supervising the training of the skeleton model. Initially, the features output by the skeleton encoder are divided into four parts and contrastively learned with the corresponding textual features generated by the text encoder. The resulting contrastive loss guides the overall network training. To achieve this, a novel loss function is designed for the training phase of the network. In the testing phase, only the loss of the skeleton model is considered, excluding the contrastive loss between the skeleton and text features from the computation. Furthermore, recognizing similar actions remains a challenging problem for skeleton-based action recognition networks. Some similar actions exhibit subtle differences in the joint coordinate motion trajectories in each frame, which increases the difficulty of recognition for the model. Traditional features like joints, bones, and velocities struggle to effectively distinguish between these similar actions. Therefore, angle features, representing high-order features, are incorporated into the skeleton model input to aid in the classification of similar actions. Moreover, a multi-feature skeleton encoder is designed to extract information separately from joints, bones, velocities, and angles, followed by feature fusion. The fused features are then passed through three graph convolution blocks before being fed into fully connected layers for classification. Additionally, a spatiotemporal attention module is introduced into each graph convolution block to enable the model to focus more on key information in the spatiotemporal region, thereby further enhancing the recognition accuracy of the network. Finally, the proposed model is evaluated against state-of-the-art methods using three skeleton-based action recognition datasets NTU RGB + D 60 [8], NTU RGB + D 120 [9], and NW-UCLA [10]. The main contributions of this paper are summarized as follows:

- By incorporating semantic information generated by large-scale language models, our approach offers a more comprehensive and enriched feature representation for action recognition tasks.
- We propose a multi-feature skeleton encoder that integrates four input branches in the early stages of the model, followed by the application of spatiotemporal graph convolution modules to extract fused features. This architecture not only preserves rich input features but also significantly reduces the complexity of the model with fewer parameters, making it easier to train.
- Extensive experiments conducted on three large-scale datasets, NTU RGB + D 60, NTU RGB + D 120, and Northwestern-UCLA, validate the effectiveness of our model.

## 2. Related Works

The graph neural network (GNN) [11,12,13,14,15] constitutes a class of deep learning models designed for processing graph data, and has emerged as a focal point of research across various domains including computer vision, natural language processing, and recommendation systems. The GNN manifests in various forms, such as the graph convolutional network (GCN) [11], the graph attention network (GAT) [12], and the graph autoencoder (GAE) [13], among others. GCN [11], akin to CNN, demonstrates robust feature learning capabilities, primarily focusing on extracting spatial features from topological graphs. However, owing to the dynamic nature of graph structures and the intricacies of frequency domain processing, the traditional GCN often encounters challenges in directly handling dynamic graphs. As a result, the spatial domain-based GCN is typically better suited for action recognition tasks [16,17,18,19].

Early methods for skeleton-based behavior recognition predominantly employed a recurrent neural network (RNN) [20,21,22,23], or convolutional neural network (CNN) [24,25,26] to capture temporal features. However, these approaches primarily focused on the temporal relationships within sequences and paid limited attention to the interactions between joints. Other techniques leveraging recurrent neural networks, such as long short-term memory (LSTM) [27] and gated recurrent unit (GRU) [28], were commonly utilized to model the temporal dynamics of skeleton sequences [29,30,31]. Nonetheless, these methodologies did not explicitly convey to the network which dimension corresponded to which joint, thus lacking semantic information regarding the relationships between joints.

To adapt the convolutional neural network (CNN) to non-Euclidean structures, Jiang et al. [32] introduced the spatial–temporal graph convolutional network (ST-GCN), which has found widespread use in behavior recognition tasks involving skeleton sequences. ST-GCN models the behavior of skeleton sequences by employing graph convolutional networks within a spatiotemporal graph framework. However, the manually defined graph topology in ST-GCN poses challenges for node aggregation, thereby impacting the recognition performance and generalization capability of the network. In response, ST-GAT [16] incorporates attention mechanisms to define aggregation functions for spatiotemporal neighboring nodes and root nodes, thereby enhancing the model’s expressive capacity.

In recent years, some other GCN-based methods have been proposed [33,34,35]. Zhou et al. [33] adopted a contrastive learning strategy to enhance the discriminative ability of skeleton representation through feature refinement modules, and dynamically calibrated the feature space using multi-level contrastive loss, effectively distinguishing similar actions (such as “writing” and “typing”). In addition, the BlockGCN proposed by Zhou et al. [34] optimizes the graph structure through a topological encoding scheme, maintains bone connectivity information through persistent homology analysis, and introduces an efficient BlockGC mechanism to improve recognition performance while reducing parameters by more than 40%. The lightweight multi-scale spatiotemporal graph convolutional network (LMSTGCN) proposed by Zheng et al. [35] captures the distance dependence between joints through multi-scale spatiotemporal graph convolution (LMSGCN), and enhances temporal feature expression by combining the extended convolution time modeling (LM TCN). At the same time, the spatiotemporal position attention (STLAtt) mechanism is introduced to focus on keyframes and joints, achieving efficient and accurate action recognition on multiple datasets.

While significant progress has been achieved in explicitly exploring semantic information in domains like machine translation [36] and image recognition [37], the same level of attention has not been given to semantic information in skeleton-based action recognition. Semantic details such as joint types and frame indices are often overlooked, despite their critical relevance to behavior recognition. Zhang et al. proposed a method to integrate advanced semantic information about joints into the network, thereby enhancing its feature representation capability and preserving essential information about the spatiotemporal structure of the body [38].

Unlike the above skeleton-based action recognition methods, our approach introduces a semantics-assisted training mechanism for the first time. By performing contrastive learning between the features output by the skeleton encoder and the semantic features generated by the text encoder, a contrastive loss is generated to guide the network training. This method not only focuses on low-level features such as joints and bones but also fully utilizes the semantic information of actions, helping the model better understand the semantic relationships of actions. At the same time, compared to traditional methods based on joint and bone features, we introduce angle features as high-order features in the multi-feature skeleton encoder, providing the model with more motion information and helping it better distinguish between similar actions. Furthermore, in our approach, through early feature fusion and multi-scale graph convolution modules, the computational complexity and the number of parameters are significantly reduced, making the model more lightweight and easier to train and deploy.

## 3. Proposed Method

**Figure 1:**
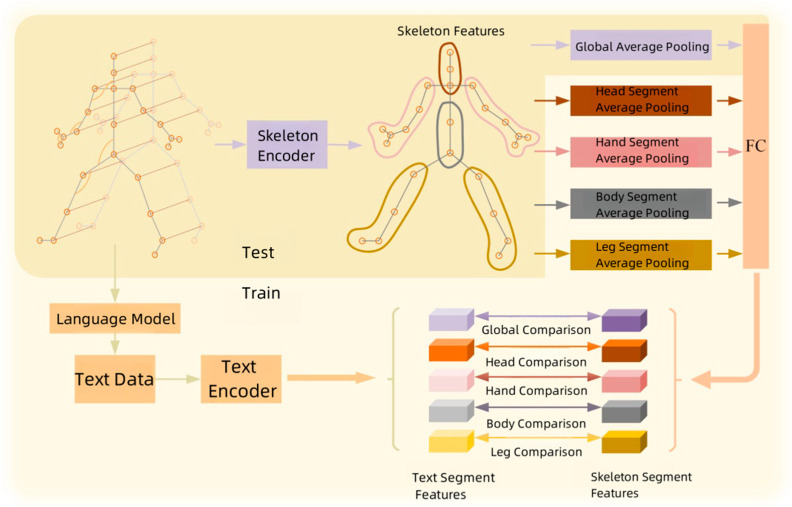
Overall network framework.

Based on the skeleton encoder and a large-scale language pre-trained model for text encoding, this paper constructs a semantics-assisted training graph convolutional network aimed at improving skeleton-based action recognition performance. The overall framework, as shown in Figure 1, consists of two main components: the skeleton model and the text encoder. Both parts participate in network training jointly, but it is worth noting that the text encoder does not directly participate in the network testing phase. The input data first passes through the skeleton encoder to generate skeleton features. These features are divided into four key parts: the head, hands, body, and legs. Subsequently, the features of each skeleton part undergo pooling and fully connected layer processing to obtain specific part features. Meanwhile, the textual descriptions of action categories are processed using the text encoder, which is based on a pre-trained large-scale language model. In the subsequent learning process, contrastive learning is applied, comparing the features of each skeleton part with the corresponding semantic text features generated by the text encoder. This helps the network better understand the correlation between skeleton features and textual descriptions, thereby improving action recognition accuracy. Overall, this paper’s approach not only focuses on fine-grained modeling of skeleton information but also integrates semantic information generated by a large-scale language model, providing a more comprehensive and enriched feature representation for action recognition tasks.

### 3.1. Preparation

Data preprocessing is considered a crucial step in skeleton-based action recognition tasks. This paper categorizes the input features, which have undergone various preprocessing techniques, into four main types: joints, skeletons, velocities, and angles. Assuming that the original three-dimensional coordinate set of an action sequence is represented by $X=\{x\in R^{C_{in}\times T_{in}\times V_{in}}$}, where $C_{in}$, $T_{in}$, and $V_{in}$ denote the input coordinates, coordinate system, and joints, respectively. Then, the relative position set is obtained as the designated positional feature $R=\{r_{i}|i=1,2,\cdots ,V_{in}\}$, where

(1) $$r_{i}=x[:,:,i]-x[:,:,c]$$

and c represents the central joint of the spine. Next, the input for joint positions is formed by concatenating X and R. Additionally, two sets of motion velocities can be obtained: $F=\left\{f_{t}|t=1,2,\cdots ,T_{in}\right\}$ for fast movements and $S=\left\{s_{t}|t=1,2,\cdots ,V_{in}\right\}$ for slow movements, using the following definitions:

(2) $$f_{t}=x[:,t+2,:]-x[:,t,:],\;s_{t}=x[:,t+1,:]-x[:,t,:]$$

By concatenating F and S for each joint, a feature vector is obtained, resulting in motion velocities. Finally, the input for skeleton features includes bone length $L=\{l_{i}|i=1,2,\cdots ,V_{in}\}$ and bone angle $A=\{a_{i}|i=1,2,\cdots ,V_{in}\}$. To obtain these two sets of features, the length and angle of each bone are calculated as follows:

(3) $$l_{i}=x\left[:,:,i\right]-x\left[:,:,i_{adj}\right],\;a_{i,w}=arccos\left(\frac{l_{i,w}}{\sqrt{l_{i,x}^{2}+l_{i,y}^{2}+l_{i,z}^{2}}}\right)$$

where $i_{adj}$ represents the adjacent joint of the i-th joint, and $w\in \left\{x,y,z\right\}$ represents the three-dimensional coordinates.

By utilizing first-order features (joint features) and second-order features (bone features), relatively high action recognition accuracy has been achieved. However, despite these achievements, certain models still tend to confuse actions with similar motion trajectories. To address this issue, this paper proposes incorporating high-order features into the skeleton model using angle encoding to more accurately capture the complex relationships between joints and body parts. In skeleton-based action recognition, third-order features are proposed to measure the angles between three body joints to describe the relative motion between body parts. Given three joints u, $w_{1}$, and $w_{2}$, where u is the target joint for computing angle features, $w_{1}$ and $w_{2}$ are endpoints in the skeleton, and $\vec{b_{u,wi}}$ represents the vector from joint u to $w_{i}$, then the following can be expressed:

(4) $$\vec{b_{u,wi}}=(x_{wi}-x_{xu},y_{wi}-y_{xu},y_{wi}-y_{xu})$$

where $\left(x_{k},y_{k},z_{k}\right)$ represents the joint coordinates of joint $k\left(k=u,w_{1},w_{2}\right)$. Assuming θ is the angle between $\vec{b_{u,w1}}$ and $\vec{b_{u,w2}}$, the static angle encoding $d_{a}\left(u\right)$ for joint u is defined as follows:

(5) $$d_{a}\left(u\right)=\left\{\begin{array}{cc} 1-\cos\theta =1-\frac{\vec{b_{u,w1}}\cdot \vec{b_{u,w2}}}{\left|\vec{b_{u,w1}}||\vec{b_{u,w2}}\right|} & if\;u\neq w_{1},u\neq w_{2}\\ 0 & if\;u=w_{1},u=w_{2} \end{array}\right.$$

Please note that $w_{1}$ and $w_{2}$ do not need to be adjacent nodes of u. The feature value θ monotonically increases as it ranges from 0 to π radians. Compared to first-order features that represent joint coordinates and second-order features that represent bone lengths and directions, these third-order features emphasize motion and are scale-invariant to the subject’s body.

**Figure 2:**
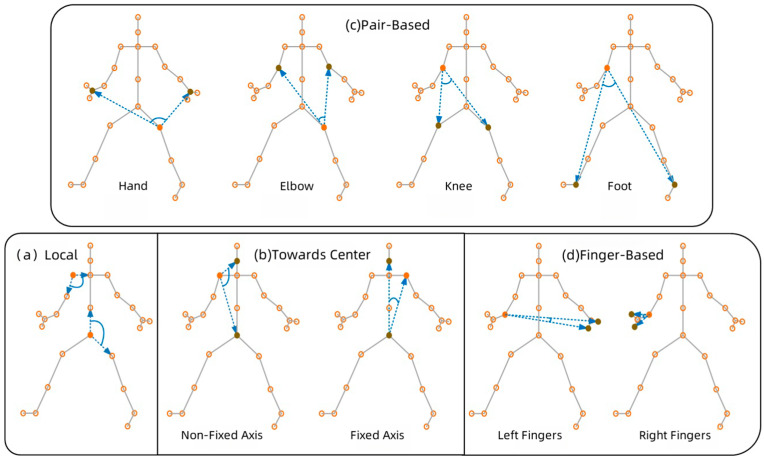
Diagram of four angle feature representations.

However, leveraging these angle features presents a computational challenge. If we choose to use all possible angles, involving all combinations between joints u, $w_{1}$, and $w_{2}$, the computational complexity reaches $O\left(N^{3}T\right)$, where N and T represent the number of joints and frames, respectively. This leads to extremely high computational costs, limiting the model’s practical feasibility. To address this issue, we adopted a strategy of manually defining key angles. These angles play a crucial role in distinguishing actions without significantly increasing computational costs. Specifically, as illustrated in Figure 2, four representative angle features are manually selected.

(a) Locally Defined Angles: As shown in Figure 2a, a locally defined angle is measured between one joint and its two adjacent joints. If a target joint has only one neighboring joint, its angle feature is set to zero. When a joint has more than two neighboring joints, the two most active ones are selected. For example, the neck joint uses the two shoulders rather than the head and abdomen, as the latter move less frequently. These angles capture the relative motion between two bones.
(b) Center-Facing Angles: This metric quantifies the angular relationship between a given target joint and the body’s central joints—specifically, the neck and pelvis. As illustrated in Figure 2b, two types of center-directed angles are defined for each target joint: (1) the angle formed by the neck, target joint, and pelvis, referred to as the non-fixed axis, and (2) the angle formed by the neck, pelvis, and target joint, known as the fixed axis. For the neck and pelvis themselves, their corresponding angle features are assigned a value of zero. Center-facing angles serve to characterize the spatial positioning of a target joint relative to the body’s central structure. For instance, when an elbow joint extends horizontally away from the body’s center, the non-fixed axis angle diminishes, whereas the fixed axis angle increases.
(c) Pair-Based Angles: This type of angle quantifies the angular relationship between a target joint and four key endpoint pairs—hands, elbows, knees, and feet—as depicted in Figure 2c. If the target joint itself belongs to these endpoints, its feature value is assigned as zero. These specific joint pairs are selected due to their crucial role in action execution. Pair-based angles play an important role in identifying object-related movements. For instance, when an individual holds a box, the angle formed between the target joint and the hand can provide insights into the box’s size.
(d) Finger-Based Angles: Fingers play an active role in human actions. When each hand’s skeletal structure includes finger joints, more detailed finger-based angles are incorporated. As shown in Figure 2d, two finger joints are chosen as anchor points to define an angle. These finger-based angles serve as an indirect representation of gestures. Finger-based angles can indirectly describe gestures. For instance, the angle with the wrist as the root, and the fingertips and thumb as two endpoints, can reflect the openness of the hand.

This strategy aims to balance computational efficiency and model performance by extracting key information that can effectively differentiate between different actions. These manually defined angle features provide targeted information to the model, helping to better capture the motion characteristics of joint movements in the skeleton structure. This enhances the effectiveness and practicality of the model.

### 3.2. Composition Architecture

**Figure 3:**
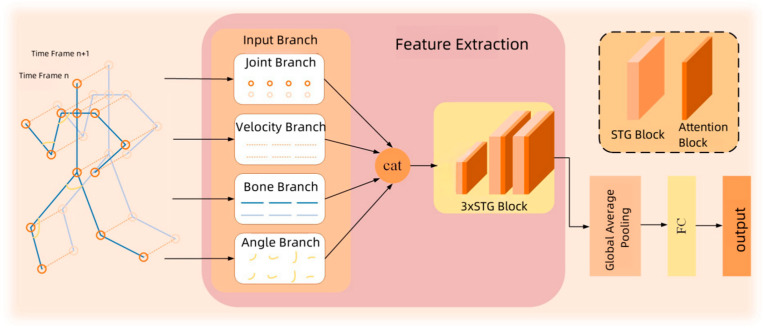
Skeleton model architecture.

Current networks predominantly utilize a multi-stream architecture [18,19,36] for decision fusion in models. However, this approach often demands a more complex computation process, higher computational resources, and longer training times, which complicates hyperparameter tuning on large-scale datasets. To address these issues, this paper introduces a multi-feature skeleton model, as depicted in Figure 3. This model extracts information on joints $X_{j}\in R^{C\times T\times V}$, skeletons $X_{b}\in R^{C\times T\times V}$, velocities $X_{v}\in R^{C\times T\times V}$, and angles $X_{a}\in R^{C\times T\times V}$ from the data. Here, C, T, and V represent the number of channels, the number of time frames, and the number of joints, respectively. These features are then fed into their corresponding branches (joint branch, skeleton branch, velocity branch, and angle branch) to extract feature information. Subsequently, they are concatenated along the channel dimension, and are represented as follows:

(6) $$I_{out}=I\left(\tilde{X}\right)=\left[{Y\left(X_{j}\right)}_{j}||{Y\left(X_{b}\right)}_{b}||{Y\left(X_{v}\right)}_{v}||{Y\left(X_{a}\right)}_{a}\right]$$

where $\tilde{X}\in R^{4\times C\times T\times V}$, $I_{out}\in R^{C^{\prime }\times T\times V}$, and $I\left(\cdot \right)$ represent the input, output, and multi-feature extraction modules of the Input Branch, respectively. The multi-feature extraction module includes three sequential spatiotemporal graph convolutional modules (STG Blocks). || represents the channel concatenation operation. $Y_{j}\in R^{C^{\prime }/4\times T\times V}$, $Y_{b}\in R^{C^{\prime }/4\times T\times V}$, $Y_{v}\in R^{C^{\prime }/4\times T\times V}$, and $Y_{a}\in R^{C^{\prime }/4\times T\times V}$ denote the outputs of the joint branch, skeleton branch, velocity branch, and angle branch within the multi-feature extraction module, respectively.

After the fusion of features from the four branches, $I_{out}$ is input into three sequential spatiotemporal graph convolutional modules. Finally, after global pooling and fully connected layers, the output $G\in R^{C^{\prime }\times T\times V}$ is obtained. This process can be represented as follows:

(7) $$G=fc\left(gap\left({STG}_{3}\left(I_{out}\right)\right)\right)$$

where $fc\left(\cdot \right)$ and $gap\left(\cdot \right)$ represent the fully connected layer and the global pooling layer, respectively. ${STG}_{3}\left(\cdot \right)$ represents the three sequential spatiotemporal graph convolutional modules. As shown in Figure 3, each spatiotemporal graph convolutional module contains a Multiscale Graph Convolution Module (MGCN) and an Attention Block.

**Figure 4:**
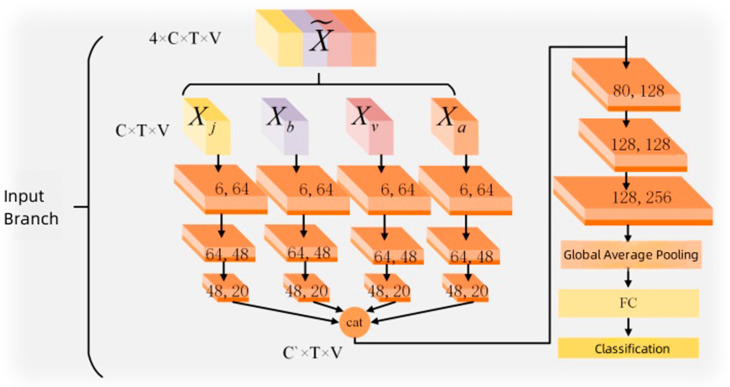
Skeleton model channel numbers.

Figure 4 illustrates the number of channels in the skeleton model, where C is 6 and $C^{\prime }$ is 80. The channels in the spatiotemporal graph convolutional modules are structured as follows: 64-48-20-128-128-256. The temporal dimension is halved after the temporal convolution in the fourth and sixth blocks. After the third block of temporal graph convolution, the features from the four branches are concatenated along the channel dimension. This means that the four input branches are fused in the early stages of the model, followed by the application of a spatiotemporal graph convolutional module to extract the fused features. This architecture not only preserves rich input features but also significantly reduces the complexity of the model, with fewer parameters, making it easier to train.

#### 3.2.1. Graph Convolutional Module

**Figure 5:**
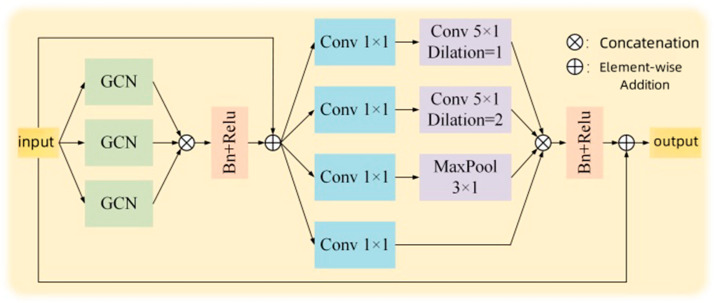
Graph convolutional model framework.

Figure 5 illustrates the framework of the multiscale graph convolutional model, which consists of spatial and temporal components, designed to comprehensively explore the topological structure and temporal features of action sequences. In the spatial component, three parallel graph convolution branches are introduced to extract features of the topological structure across different channels. This design enriches the representation of the topological structure, enhances the correlation between nodes, and improves the model’s sensitivity to spatial information. These three branches perform feature fusion through channel concatenation, reducing the parameter count of the model compared to element-wise addition, thereby decreasing training time and improving efficiency. In the temporal component, a multiscale temporal convolutional model is employed with different dilation rates. This strategy enables the model to effectively capture action information at various temporal scales, thereby enhancing its ability to recognize long-duration action categories. By introducing multiscale convolutions in the temporal domain, the model’s temporal modeling capability is further enhanced, making it more adaptive and robust. The process of a single graph convolution in Figure 5 is represented as follows:

(8) $$F_{s}=\sum_{i=1}^{c}M\left(\varphi (X_{i})-\phi (X_{i})+\partial \hat{A_{s}}\right)X_{i}W$$

Here, $F_{s}$ represents the output of a single graph convolution, $M\left(\cdot \right)$ denotes the topological modeling function, and $\varphi \left(\cdot \right)$ and $\phi \left(\cdot \right)$ are linear transformation functions (utilizing 1 × 1 convolutions). Additionally, ∂ is a trainable scalar used to adjust the topological structure, while W represents the weight of the graph convolution. In this context, $\hat{A_{s}}=\Lambda_{s}^{-1/2}A_{s}\Lambda_{s}^{-1/2}$, $\Lambda_{s}^{ij}=\sum_{j}\left(A_{s}^{ij}\right)$, and $S=\left\{root,centripetal,centrifugal\right\}$ define the neighborhoods of each joint node, where root is the self node, centripetal denotes the nodes close to the skeleton center, and centrifugal represents the nodes away from the center.

#### 3.2.2. Attention Module

In previous skeleton-based action recognition methods, attention modules were typically implemented using multilayer perceptron (MLP), including some classical structures such as AGC-LSTM [39] and MS-AAGCN [18]. These modules are characterized by independently applying the attention mechanism to each channel or spatial dimension, while other dimensions are globally average-pooled into a single unit. Specifically, MLP structures are used to model each channel or spatial position, capturing the complex relationships and features within the input skeleton data. For each channel or joint node, an independent MLP generates a weight to adjust the importance of that channel or joint node.

**Figure 6:**
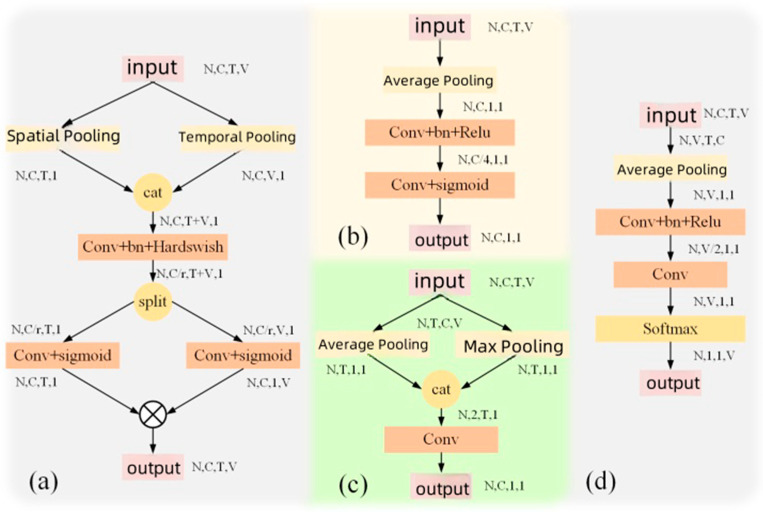
Four attention models. (**a**) shows the proposed framework for spatio-temporal joint attention, (**b**) is the structural diagram of channel attention, (**c**) is the structural diagram of temporal attention, (**d**) is the structural diagram of joint attention.

As shown in Figure 6b–d, channel attention (ca), temporal attention (fa), and joint attention (ja) are respectively demonstrated. These attention mechanisms pool the attention weights down to a scalar by pooling over the temporal and spatial dimensions. However, intuitively, the spatiotemporal information of actions may be interrelated, and considering attention separately for frames and joints may not capture this correlation. In reality, a moment in an action may depend on specific combinations of joints and their temporal relationships.

To address this issue, this paper introduces spatiotemporal joint attention (stja) to jointly discern the specific joints that carry the most informative signals across frames of the entire skeleton sequence. As illustrated in Figure 6a, the framework for spatiotemporal joint attention is presented. First, the input features are averaged over both the temporal and spatial dimensions. Subsequently, these concatenated feature vectors are passed through convolutional layers to compress the information. Then, two independent convolutional layers are utilized to obtain two sets of attention scores for the temporal and spatial dimensions. Finally, the scores from both temporal and spatial dimensions are multiplied by the outer product along the channel direction, yielding attention scores for the entire action sequence. By jointly modeling spatiotemporal information, richer attention weights are provided, thereby enhancing the recognition performance of action categories. The proposed spatiotemporal joint attention module can be expressed as follows:

(9) $$f_{inner}=\theta \left(({pool}_{t}(f_{in})⊕{pool}_{v}(f_{in}))\cdot W\right),\;{f_{out}=f}_{in}⊙\left(\sigma (f_{inner}\cdot W_{t})⊗\sigma (f_{inner}\cdot W_{v})\right)$$

where $f_{in}$ and $f_{out}$ represent the input and output feature maps, ⨁ denotes the concatenation operation, ⨂ and ⨀ represent channel-wise multiplication and element-wise dot product, ${pool}_{t}\left(\cdot \right)$ and ${pool}_{v}\left(\cdot \right)$ are average pooling operations over the temporal and spatial dimensions, $\theta \left(\cdot \right)$ and $\sigma \left(\cdot \right)$ represent Sigmoid and HardSwish activation functions, and $W\in R^{C\times C/r}$, $W_{t}\in R^{C/r\times C}$, and $W_{v}\in R^{C/r\times C}$ are trainable weight parameters.

### 3.3. Text Encoder

In this work, we use large-scale language models (e.g., GPT) to generate textual descriptions of actions. To generate the required action descriptions, three text prompting strategies are designed:

- Paragraph: This strategy provides a detailed paragraph describing the entire action comprehensively.
- Synonyms: It gathers 10 synonyms for each action label.
- Partial Description: For each action, it collects information describing different body parts.

The skeleton partitioning strategy is illustrated in Table 1. Taking ‘drink water’ and ‘brush teeth’ as examples, Table 1 shows the prompts used to generate different descriptions.

Considering the recent success of transformer models in NLP, transformers are employed to design the text encoder in this paper. Assuming the text encoder consists of L encoders, each encoder comprises a self-attention mechanism $\left(SA\right)$, a normalization layer $\left(LN\right)$, and a feed-forward neural network layer $\left(FFN\right)$. The transformer encoder can be represented as follows:

(10) $$\hat{z_{l}}=SA\left(LN\left(z_{l-1}\right)\right)+z_{l-1},\;z_{l}=FFN\left(LN\left(\hat{z_{l}}\right)\right)+\hat{z_{l}}$$

where $\hat{z_{l}}$ and $z_{l}$ respectively represent the output features of modules SA and FFN in module l. The computation of SA is as follows:

(11) $$SA\left(Q_{l},K_{l},V_{l}\right)=softmax\left(\frac{Q_{l}K_{l}^{T}}{\sqrt{d}}V_{l}\right)$$

where $Q_{l}$, $K_{l}$, and $V_{l}$ represent the query matrix, key matrix, and value matrix of module l, computed through a linear mapping by $z_{l-1}$, with weights $W_{l}^{Q}$, $W_{l}^{K}$, and $W_{l}^{V}$ respectively. d is the scaling factor, with a value equal to the dimensionality of the query and key matrices.

### 3.4. Loss Function Design

Unlike single-label supervision in skeleton classification, skeleton-language contrastive learning incorporates natural language as supervision. It adopts a dual-encoder architecture, where the skeleton encoder $E_{s}$ processes skeleton data, and the text encoder $E_{t}$ encodes action descriptions. These two encoders are jointly trained by contrasting skeleton-text pairs in both directions within each batch:

(12) $$p_{i}^{st}\left(s_{i}\right)=\frac{exp\left(sim\left(s_{i},t_{i}\right)/\tau \right)}{\sum_{j=1}^{N}exp\left(sim\left(s_{i},t_{i}\right)/\tau \right)},\;p_{i}^{ts}\left(t_{i}\right)=\frac{exp\left(sim\left(t_{i},s_{i}\right)/\tau \right)}{\sum_{j=1}^{N}exp\left(sim\left(t_{i},s_{i}\right)/\tau \right)}$$

where $s=E_{s}\left(S\right)$ and $t=E_{t}\left(T\right)$ are the encoded features of the skeleton and text, $sim\left(s,t\right)$ is the cosine similarity between s and t, τ is the temperature parameter, and N is the batch size. Unlike CLIP [40], which employs a one-to-one mapping for image-text pairs, this framework allows for multiple positive matches. Consequently, KL divergence is adopted as the skeleton-text contrastive loss instead of cross-entropy loss.

(13) $$L_{con}=\frac{1}{2}E_{s,t}\sim D\left[KL\left(p^{st}\left(s\right),y^{st}\right)+KL\left(p^{ts}\left(t\right),y^{ts}\right)\right]$$

where $y^{st}$ and $y^{ts}$ are the true similarity scores.

Considering the priors of various body parts, the skeleton can be divided into multiple groups. Applying the contrastive loss to both local part features and global features, a multi-part contrastive loss is proposed. As illustrated in Figure 1, joint features within the same group are aggregated to generate representations of each part. Subsequently, part features can be obtained through corresponding part pooling. The multi-part contrastive loss function can be represented as follows:

(14) $$L_{mul}=\frac{1}{K}\sum_{k=1}^{K}L_{con}^{k}$$

where K denotes the number of parts, with the skeleton features are divided into four groups.

The overall loss function with multi-part contrastive loss can be represented as follows:

(15) $$L_{total}=L_{cls}+{\lambda L}_{mul}$$

where λ is the balancing parameter used to balance the contributions of the skeleton encoder and the text encoder losses in the overall loss, thereby adjusting their weight relationship. $L_{cls}$ is the loss function of the skeleton model. Here, cross-entropy loss is used for training the skeleton model, represented as follows:

(16) $$L_{cls}{=-ylogp}_{\theta }\left(x\right)$$

Here, y represents the real similarity score label, x denotes the input data, and $p_{\theta }\left(x\right)$ stands for the predicted probability distribution.

## 4. Experiment

In this section, experiments were conducted on three public datasets: NTU RGB + D 60, NTU RGB + D 120, and Northwestern-UCLA. The specific implementation of the experiments is as follows: Firstly, model ablation experiments were conducted using cross-subject evaluation benchmarks on the NTU RGB + D 60 dataset, demonstrating model accuracy, and complexity. Secondly, visualizations of confusion matrices on the NTU RGB + D 60 dataset were presented. Finally, comparisons were made between the proposed method and state-of-the-art models on each of the three datasets.

### 4.1. Dataset

The NTU RGB + D 60 dataset is a pivotal resource in action recognition research, offering a diverse range of skeletal data for analysis. It contains 56,880 samples spanning 60 categories. These samples, performed by 40 individuals, were meticulously captured by three cameras, ensuring a rich variety of actions from different viewpoints. Each sample comprises 25 human joints, recorded as 3D coordinates. The dataset evaluation includes two crucial benchmarks: the cross-view (X-view) and cross-subject (X-sub) benchmarks. These benchmarks assess the model’s ability to generalize across different viewpoints and individuals. In the X-view benchmark, training data are sourced from cameras 2 and 3, with testing data from camera 1. Conversely, the X-sub benchmark utilizes training data from 20 subjects, with testing data from an additional 20 subjects.

The NTU RGB + D 120 dataset is a significant expansion of the NTU RGB + D 60 dataset, offering a vast collection of 3D skeletal data for human action recognition. With 113,945 skeleton clips spread across 120 classes, this dataset provides an extensive representation of various actions. The movements were performed by three individuals and captured by an impressive array of 106 cameras, ensuring comprehensive coverage of actions from multiple viewpoints. This dataset introduces two distinct validation benchmarks: across subjects (X-sub) and across settings (X-set). In the X-sub benchmark, training data are sourced from 53 subjects, while testing data are sourced from the remaining 53 subjects. As for the X-set benchmark, training data are derived from samples with even collector ids, whereas test data are sourced from samples with odd ids.

The Northwestern-UCLA dataset, captured by a Kinect camera, is a widely used skeleton dataset in the field of action recognition. It consists of 1494 skeleton sequences categorized into 10 classes. Notably, these actions were performed by 10 different subjects, ensuring a diverse range of movements. The evaluation setup mirrors the cross-view benchmark used in the NTU RGB + D 60 dataset. Training data are sourced from cameras 1 and 2, while test data are exclusively from camera 3.

### 4.2. Implementation Details

The experiments were conducted using the PyTorch.2.3.0 platform on a single GPU 3090. For both NTU RGB + D and NTU RGB + D 120 datasets, each sample was resized to 64 frames. The text encoder was based on CLIP, and its parameters were fine-tuned during training. The temperature setting for the contrastive loss was 0.1. For NTU RGB + D, the model was trained for 120 epochs with a batch size of 100. For NTU RGB + D 120, the model was trained for 140 epochs with a batch size of 64. A warm-up strategy was employed for the first 5 epochs. The initial learning rate was set to 0.1, with reductions by a factor of 10 at the 35th and 80th epochs. The weight decay was set to 0.00005. For NW-UCLA, the batch size, epochs, learning rate, weight decay, decay steps, and warm-up stages were set to 64, 110, 0.2, 0.00004, [90,100], and 5, respectively.

### 4.3. Ablation Experiment

To highlight the effectiveness of the lightweight skeleton action recognition network proposed in this paper, extensive experiments were conducted on the NTU RGB + D 60 dataset. Firstly, various components of the proposed model were tested to validate the effectiveness of each component, as shown in Table 2, Table 3 and Table 4. Additionally, models with different input data modalities were constructed, namely: joints, skeletons, velocities, and angles. Subsequently, the accuracy of different data modality combinations was calculated separately, as shown in Table 5.

In Table 2, “4s” represents the skeleton model with early fusion of data from four modalities, “MGCN” denotes the multi-scale graph convolutional module, “AT” stands for the spatiotemporal attention module, and “lst” indicates the semantics-assisted training framework. Under the X-sub benchmark of the NTU-RGB + D dataset, replacing the baseline model with the 4s skeleton model, MGCN module, AT module, and lst semantics-assisted training framework individually resulted in the highest accuracy (increased by 1.1%) and the lowest parameter count (reduced by 2M) when all were replaced by the models presented in this paper. This highlights the effectiveness of the 4s skeleton model, MGCN module, AT module, and lst semantics-assisted training framework. Using 4s instead of the baseline’s skeleton model led to a reduction of 0.21M parameters, demonstrating the lightweight nature of the early fusion framework proposed in this paper. Additionally, adding the MGCN and AT modules on top of 4s resulted in 1% accuracy increase and a 2M parameter reduction, confirming the effectiveness of the multi-scale graph convolutional module and the spatiotemporal attention module.

As shown in Table 3, “ja”, “ca”, “fa”, and “stja” represent the joint attention, channel attention, temporal attention, and spatiotemporal joint attention modules, respectively. Under the X-sub benchmark of the NTU-RGB + D dataset, the comparison of model accuracy and parameter count when incorporating the four proposed attention modeling modules into the network (without semantics-assisted training) is presented. From the table, it is evident that when using the stja attention module, the model achieves the highest accuracy, indicating the effectiveness of the spatiotemporal joint attention module proposed in this paper.

In Table 4, the experimental results under the X-sub benchmark of the NTU-RGB + D dataset (without semantics-assisted training) are presented. “Int” indicates the number of layers used in the input module (i.e., the stage of the four branches), and “STG” represents the number of layers after fusion. From the table, it can be observed that when using three layers of spatiotemporal graph convolution modules in the four-branch stage and three layers of graph convolution modules after fusion, the accuracy of the proposed model in this paper is 90.5%, with a minimum parameter count of 0.75 M. Although increasing the number of layers leads to higher model accuracy, it also results in an increase in model parameters. To reduce the parameter count of the proposed model, using three layers of spatiotemporal graph convolution modules in the four-branch stage and three layers of graph convolution modules after fusion achieves a balance between model accuracy and parameter count. This approach enables the model to achieve lightweightness while effectively classifying actions.

As shown in Table 5, the results of fusion under different combinations of data modalities, using the optimal configurations, are presented. From the table, it can be observed that when early fusion is performed using all four features, the model achieves the highest accuracy of 90.9%. When only joint and skeletal features are used, the accuracy is 89.1%. However, adding angles on top of joint and skeletal features increases the accuracy to 90.1%, resulting in a 1% improvement compared to the previous configuration. This demonstrates the effectiveness of the proposed angle encoding method in this paper.

### 4.4. Visualization

**Figure 7:**
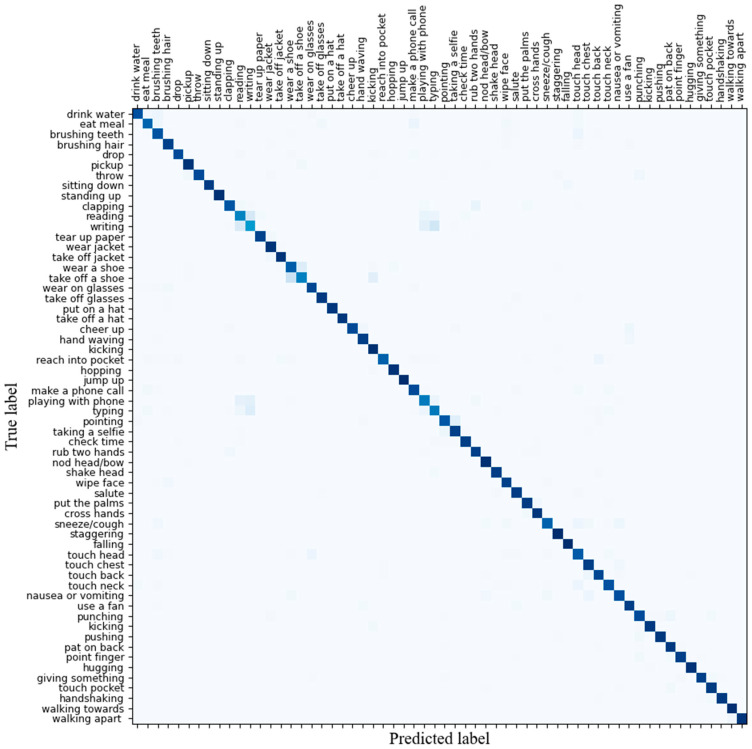
Confusion matrix on the NTU RGB + D dataset.

Figure 7 visualizes the experimental results, illustrating the confusion matrix of the proposed model on the NTU RGB + D dataset. The results depicted in the figure demonstrate the superior recognition performance of the proposed model.

### 4.5. Comparison with Other Methods

To demonstrate the effectiveness of the proposed model, comparisons were made with state-of-the-art methods on three public datasets: NTU RGB + D, NTU RGB + D 120, and Northwestern-UCLA. Additionally, a comparison of the model’s parameter count was performed on the NTU RGB + D dataset to highlight the model’s lightweight nature. To ensure a comprehensive comparison, the parameter count metric is based on the results reported by EfficientGCN [41]. The compared models include GCN-based methods such as [5,6,17,18,33,34,35,42,43,44,45,46], CNN-based methods such as [47], and RNN-based methods such as [37,48]. The comparison results of the proposed model are shown in Table 6, Table 7 and Table 8.

As shown in Table 6, on the X-sub and X-view benchmarks of the NTU-RGB + D dataset, the proposed model is only 1.9% and 0.1% lower than Zhou et al. [33] and LMSTGCN [35], respectively, on the X-sub benchmark, and only 0.6% lower than Zhou et al. [33] on the X-view benchmark. But our model has less than half the number of parameters compared to these two methods. Compared to other methods, this model has significantly improved recognition accuracy on both X-sub and X-view benchmarks. For instance, compared to MSSTNet [47], the model achieves an increase of 1.3% and 0.9% on the X-sub and X-view benchmarks, respectively. Among GCN-based methods, compared to ST-GCN, 2s-AGCN [6], and AS-GCN [17], the model shows improvements of 9.4%, 1.4%, and 4.1% on the X-sub benchmark, and 7.9%, 1.1%, and 7.5% on the X-view benchmark, respectively. In terms of model parameter count, the proposed model has the fewest parameters compared to all these methods. Since some methods have not disclosed their computational complexity and model code, we are unable to obtain their computational complexity. However, compared to methods with known computational complexity, our model also has the least complexity. Overall, despite the lower parameters and computational complexity, the proposed model is still able to maintain high recognition accuracy compared to most methods. It strikes a good balance between recognition efficiency and accuracy, effectively controlling model complexity while ensuring strong recognition ability, demonstrating superior overall performance.

In Table 7, the model achieves recognition accuracies of 87.3% and 88.9% on the X-sub and X-set benchmarks of the NTU-RGB + D 120 dataset, respectively. The results demonstrate that the proposed model outperforms most methods, showing a 16.6% improvement over the ST-GCN method on the X-set benchmark and a 15.7% improvement on the X-sub benchmark. Compared to CNN-based methods, the model achieves improvements of 2% and 2.9% on the X-sub and X-set benchmarks, respectively, compared to MSSTNet [47]. These comparative results validate the superior classification performance of the model on large-scale datasets.

As presented in Table 8, the model achieves superior recognition performance on the Northwestern-UCLA dataset. When compared to RNN-based approaches, the model achieves a 5.4% and 1.3% improvement in recognition accuracy over the Ensemble TS-LSTM [48] and 2s-AGC-LSTM [39] methods, respectively. Among GCN-based methods, the model shows a 5% and 0.7% improvement in recognition accuracy compared to the 2s-AGCN [6] and IA-ASGCN [43] methods, respectively. The comparative results across the three datasets demonstrate the model’s superior recognition performance across various dataset scales, indicating strong generalization capabilities. Additionally, the model achieves a significant reduction in parameters compared to other models on the NTU-RGB + D dataset, demonstrating its lightweight nature while maintaining high recognition performance.

## 5. Conclusions

The proposed SAT-GCN addresses two key challenges: the large parameter size of existing models and the difficulty in recognizing action categories with similar motion trajectories. Firstly, a text encoder is introduced to extract textual descriptions of action categories, which are then used to supervise the training of the skeleton model. During training, contrastive learning is employed by splitting the output of the features by the skeleton encoder and those generated by the text encoder into five parts. This contrastive loss guides the overall network training process. At the testing stage, only the loss of the skeleton model is considered, while the contrastive loss between skeleton and text features is ignored. To tackle the challenge of recognizing similar actions, angle features are introduced into the input of the skeleton model as higher-order feature representations. These angle features assist the model in better classifying similar actions. Additionally, a multi-feature skeleton encoder is designed to independently extract joint, skeleton, velocity, and angle features, which are then fused. The fused features are passed through three GCN blocks and fed into fully connected layers for classification. Moreover, spatial–temporal attention modules are incorporated into each GCN block to enhance the model’s focus on crucial spatial–temporal information, thereby further improving action recognition accuracy.

The effectiveness of the proposed network design was thoroughly validated through extensive experiments on three large-scale datasets: NTU RGB + D, NTU RGB + D 120, and Northwestern-UCLA. On the NTU RGB + D dataset, the SAT-GCN achieved an accuracy of 90.9% with a parameter count of 0.75 million. On the NTU RGB + D 120 dataset, the network achieved an accuracy of 87.3%. Lastly, on the Northwestern-UCLA dataset, the network achieved an accuracy of 94.6%. These results demonstrate the efficacy of the proposed approach across different datasets and underscore its potential for action recognition tasks.

While the proposed SAT-GCN has shown promising results, it requires significant computational resources for training and a larger amount of training data to improve recognition performance. Future research should explore how to achieve similar recognition performance with fewer training samples, aiming to enhance the model’s efficiency and applicability.

## Figures and Tables

**Table 1:** Text Descriptions Generated from Different Prompt Inputs.

Paragraph	Synonyms	Partial Description
drink water	drink water	drink water
The man is drinking water from a glass. He is holding the glass and bringing it to his mouth. He tilts the glass back and forth, letting the water flow into his mouth. He is swallowing the water and then places the glass on the table.	sip, guzzle, gulp, swig, chug, quaff, swill, slug, chug down, toss back.	with the head slightly tilted backward; holding the cup with the hand; hip remains stationary; leg remains stationary.
brush teeth	brush teeth	brush teeth
The man is standing in front of the sink, looking in the mirror as he brushes his teeth. He is wearing a blue shirt and jeans, and his hair is wet from a recent shower. He is methodically brushing his teeth, making sure to get every tooth clean. He spits the toothpaste into the sink and rinses his mouth out with water. He then puts his toothbrush away and washes his hands.	scrub teeth, clean teeth, polish teeth, rinse teeth, gargle, floss, brush gums, brush tongue, clean palate, clean throat.	The head is slightly tilted forward; hand brings toothbrush up to mouth; hip remains stationary; leg remains stationary.

**Table 2:** Comparison of model validation accuracy and parameter count under different configurations. ↓ represents a decrease compared to the baseline, ↑ represents an increase compared to the baseline.

Models	Accuracy (%)	Params (M)
baseline	89.5	2.75
+4s	89.2 (0.3↓)	0.68 (2.07↓)
+MGCN	89.8 (0.3↑)	1.45 (1.3↓)
+AT	89.7 (0.2↑)	2.86 (0.11↑)
MGCN + AT	90.0 (0.5↑)	1.56 (1.19↓)
4s + MGCN + AT	90.5 (1↑)	0.75 (2↓)
Ours (4s + MGCN + AT + lst)	90.9 (1.4↑)	0.75 (2↓)

**Table 3:** Comparison of accuracy under different attention modules.

Attention Module	Accuracy (%)	Params (M)
ja	90.2	0.61
ca	90.2	0.58
fa	90.3	0.53
stja	90.5	0.75

**Table 4:** Comparison of model accuracy and parameter count for different layer configurations.

Models	Accuracy (%)	Params (M)
4Int + 3STG	90.5	0.75
4Int + 4STG	90.5	0.89
4Int + 5STG	90.5	1.12
3Int + 3STG	90.5	0.75
3Int + 4STG	90.0	1.00
3Int + 5STG	90.5	1.3

**Table 5:** Comparison of accuracy for different data modality combinations.

Data Modality	Accuracy (%)
Joint + Skeleton	89.1
Joint + Velocity	88.7
Joint + Skeleton + Velocity	89.5
Joint + Angle	89.8
Joint + Skeleton + Angle	90.1
4s (Joint + Skeleton + Velocity + Angle)	90.9

**Table 6:** Comparison of recognition accuracy with state-of-the-art methods on the NTU-RGB + D dataset.

Methods	NTU RGB + D		Params (M)	FLOPs (G)
	X-Sub (%)	X-View (%)		
ST-GCN [5]	81.5	88.3	3.10	16.30
2s-AGCN [6]	88.5	95.1	6.94	37.30
DCA-SGIN [42]	87.2	88.7	-	-
AS-GCN [17]	86.8	94.2	9.50	6.10
MS-AAGCN [18]	90.0	96.2	15.04	74.80
FLAGCN [44]	89.4	94.8	0.83	4.10
DGNN [46]	89.9	96.1	26.24	-
Dynamic GCN [49]	91.5	96.0	14.40	1.99
MSSTNet [47]	89.6	95.3	39.60	-
Zhou et al. [33]	92.8	96.8	1.99	-
BlockGCN [34]	90.9	95.4	1.30	1.63
LMSTGCN [35]	91.0	95.8	5.40	-
Ours	90.9	96.2	0.75	0.78

**Table 7:** Comparison of recognition accuracy with state-of-the-art methods on the NTU-RGB + D 120 dataset.

Methods	NTU RGB + D 120	
	X-Sub (%)	X-Set (%)
ST-GCN [5]	70.7	73.2
2s-AGCN [6]	82.5	84.2
AS-GCN [17]	77.9	78.5
IA-ASGCN [43]	85.4	87.4
DC-GCN [45]	87.1	88.6
MSSTNet [47]	85.3	86.0
Ours	87.3	88.9

**Table 8:** Comparison of recognition accuracy with state-of-the-art methods on the Northwestern-UCLA dataset.

Methods	Northwestern-UCLA
	Accuracy (%)
Ensemble TS-LSTM [48]	89.2
2s-AGC-LSTM [39]	93.3
2s-AGCN [6]	89.6
IA-ASGCN [43]	93.9
Ours	94.6

## Data Availability

Data will be provided by the corresponding author upon reasonable request by the reader.
